# Dentigerous Cyst associated with Horizontally Impacted Mandibular Second Premolar

**DOI:** 10.5005/jp-journals-10005-1235

**Published:** 2014-04-26

**Authors:** Rahul Mishra, Abhay Mani Tripathi, Monika Rathore

**Affiliations:** Senior Lecturer, Department of Pedodontics and Preventive Dentistry Purvanchal Institute of Dental Sciences, Gorakhpur, Uttar Pradesh, India; Reader, Department of Pedodontics and Preventive Dentistry, Sardar Patel PG Institute of Dental and Medical Sciences, Lucknow Uttar Pradesh, India; Professor, Department of Pedodontics and Preventive Dentistry, BBD College of Dental Sciences, Lucknow, Uttar Pradesh, India

**Keywords:** Odontogenic infections, Dentigerous cyst, Impaction, Surgical pedodontics

## Abstract

Dentigerous Cyst/developmental cyst of benign odontogenic origin are ones that surround the crown of impacted, embedded, unerupted or developing teeth. Dentigerous cyst is second most common cyst of the oral cavity after radicular cyst. They are usually solitary in occurrence and mostly associated with the mandibular third molars. Dentigerous cysts involving impacted second premolars are rarely reported in the literatures. We present a rare case of dentigerous cyst in a 12-year-old female patient associated with an impacted mandibular second premolar.

**How to cite this article: **Mishra R, Tripathi AM, Rathore M. Dentigerous Cyst associated with Horizontally Impacted Mandibular Second Premolar. Int J Clin Pediatr Dent 2014;7(1): 54-57.

## INTRODUCTION

Dentigerous Cyst/developmental cyst of odontogenic origin are ones that surround the crown of impacted, embedded, unerupted or developing teeth. Dentigerous cyst is second most common cyst of the oral cavity after radicular cyst.

Incidence of dentigerous cyst is 20 to 24% of the entire epithelial lined jaw cyst. Frequency of 1.44% cyst for every 100 unerupted teeth in general population with more frequently occurs during 2nd – 3rd decades of life. Also occurs in children and adolescence during mixed dentition period. Affects males more frequently than females and is seen most commonly around the crowns of mandibular third molars, maxillary canines and maxillary third molars.

## CASE REPORT

A 12-year-old girl reported to the Department of Pedo-dontics and Preventive Dentistry, Babu Banarasi Das College of Dental Sciences, Lucknow with a chief complaint of a painful swelling in the lower left back region of jaw. On general examination, no abnormality detected.

**Fig. 1:**
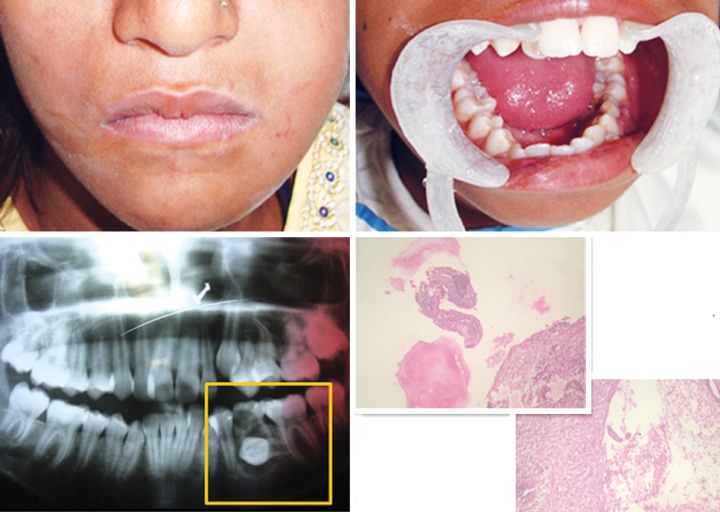
Preoperative photographs and investigations

On extraoral examination, asymmetrical face with swelling on lower left side of face and palpable and tender left submandibular lymph node (Fig. 1).

On intraoral examination, grossly carious mandibular left deciduous second molar with circumscribed swelling. Swelling extended from distal side of deciduous first molar to mesial side of permanent first molar along the vestibule. On palpation swelling was painful and firm (*see *Fig. 1).

On radiographic examination, OPG reveals a well defined unilocular radiolucent area characterized by sclerotic boarder in the region of deciduous second molar surrounding the crown of horizontally impacted left second premolar. The dentigerous cyst in OPG is of central variety because the crown is enveloped symmetrically. The follicular space is 19.0 mm that shows dentigerous cyst when the space is more than 5 mm (*see *Fig. 1).

On histopathologic examination of the aspiration biopsy showed a cystic lesion, and presumptive diagnosis of the dentigerous cyst was made (*see *Fig. 1).

**Figs 2A to C:**
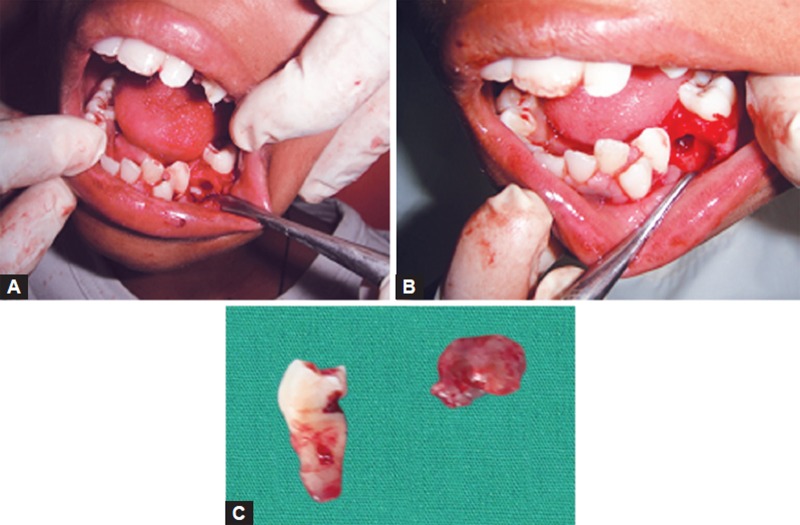
Intraoperative photographs: (A) After extraction of deciduous second molar, (B) after removal of horizontally impacted premolar, (C) cystic lining separated from the tooth

**Fig. 3:**
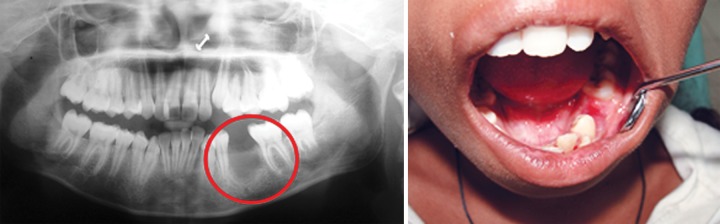
Postoperative radiograph and photographs

Surgical enucleation of the cyst to marsupialization with decompression of cyst via fenestration may be treatment of choice. In the present case, extraction of mandibular left deciduous second molar followed by surgical enucleation of the cyst was done and fragment was given for biopsy. Cystic sac enclosed the second premolar tooth firmly at the cervical margin (Figs 2A to C and 3).

## DISCUSSION

Dentigerous cysts are developmental cyst of odontogenic origin and the most prevalent, comprising 14 to 24% of the entire jaw cyst.^1,2^ Since cysts can attain considerable size with minimal or no symptoms, early detection and removal of the cysts is important to reduce morbidity. Although evidence in the literature suggests that dentigerous cysts occur more frequently during the second decade of life,^3,4^these lesions can also be found in children and adolescents. The incidence of dentigerous cysts is twice as high in male patients compared to female counterparts in contrast to the present case.^5,6^ Whites are more affected than blacks.^7^

Dentigerous cysts are usually solitary, slow growing, asymptomatic lesions that are incidentally found during routine radiographs taken to identify a missing tooth. They can occur at any location of the jaw but frequently seen in relation to impacted mandibular third molars followed by the maxillary canines and maxillary third molars.^7,8,10^Occasionally these cysts become painful when infected causing swelling and erythema. The cyst is usually small but when large, results in the expansion and thinning of the cortex leading to pathological fracture.^7,9^ Although the clinical presentations are classical of a dentigerous cyst, in our case it is associated with horizontally impacted mandibular second premolar which has not been reported.

Radiographic features are specific to the lesion charac­terized by a well defined radiolucency circumscribed by a sclerotic border, associated with the crown of an impacted or unerupted tooth. The borders may be ill-defined when infected. Rarely may they be found with odontoma or a supernumerary tooth.^8,9^ Although they mimic a normal tooth follicle, literatures suggest any follicular space of more than 4 mm to be a dentigerous cyst.^7^ Radiographically the cyst is classified according to its relation with the involve tooth crown as central, lateral and circumferential type. The central type is the most common and presents surrounding the crown. The lateral dentigerous cyst is that, which partially surrounds the crown and extends along the side of the root. The circumferential variant surrounds both the crown and the root of the involved tooth.^8^

Histologically, the lumen is lined by 2 to 4 cell layers of cuboidal to flattened nonkeratinized epithelial cells but may form keratin by metaplasia.^11^ The epithelium may be hyperplastic with the presence of hyaline bodies associated with inflammation. The connective tissue is more collagenous when infamed and contain varying degree of chronic infammatory cell infltration.^7,9^ Occasionally the cyst lining may contain ciliated and mucous secreting cells.^7^ Dentigerous cysts are treated most commonly by Enucleation,^2^ Marsupialization^13^ and decompression of cyst by fenestration.^4^ Motamedi et al suggested the criteria for selecting the treatment modality based on the age, size, location, stage of root development, position of the involved tooth and relation of the lesion to the adjacent tooth and vital structure.^12^ The most preferred treatment is enucleation with the removal of unerupted or impacted tooth. If the cyst is associated with the canine or premolar with favorable eruptive position, then extraction of the associated tooth is deferred. Large dentigerous cyst may be treated with marsupialization followed by enucleation. The prognosis is excellent when the cyst is enucleated in toto and recurrence is rare. As the lining epithelium has the pluripotential capacity, these lesions may progress to ameloblastoma, mucoepidermoid carcinoma and squamous cell carcinoma.^7^

## CONCLUSION

Dentigerous cyst associated with an impacted mandibular second premolar is extremely rare. As the clinical finding of unerupted tooth may be the only presenting symptom of a dentigerous cyst, a thorough radiographic evaluation is mandatory for all unerupted teeth that have well past their expected eruption date. Serial panoramic flms are a common and reliable tool for the diagnosis and assessment of progress in healing of space occupying lesions of jaw bones.

Why this paper is important to pediatric dentists

- Healing and bone regeneration is excellent in pediatric patients.
- Serial panoramic flms is a common and reliable tool for the diagnosis and assessment of progress in healing of space occupying lesions of jaw bones.
- Early diagnosis and being able to differentiate a develop­mental cyst from infammatory cyst is crucial in pediatric patients for subsequent treatment planning to reduce potential morbidity.
